# Abortion access in the Americas: a hemispheric and historical approach

**DOI:** 10.3389/fpubh.2023.1284737

**Published:** 2023-12-06

**Authors:** Cassia Roth

**Affiliations:** ^1^Department of Biostatistics and Epidemiology, College of Public Health, University of Georgia, Athens, GA, United States; ^2^Department of History, Franklin College of Arts & Sciences, University of Georgia, Athens, GA, United States; ^3^Latin American and Caribbean Studies Institute, Franklin College of Arts & Sciences, University of Georgia, Athens, GA, United States

**Keywords:** Latin America, Caribbean, United States, abortion, *Roe v. Wade*, reproductive justice, feminism, human rights

## Abstract

This perspective article situates the 2022 United States (U.S.) Supreme Court’s overturning of *Roe v. Wade* (1973) within the broader history of abortion rights activism and legislation in the greater Americas. The U.S. public has stereotyped Latin America and the Caribbean (LAC) as socially conservative regarding gender issues and anti-reproductive rights. But twenty-first-century LAC presents a more complicated landscape than this dominant narrative suggests. In the past 15 years, political, legislative, and public health advances and setbacks across the region provide both a blueprint for re-establishing access to safe and legal abortion and a warning on the consequences of the criminalization of abortion for the U.S. Employing a narrative approach that summarizes recent interdisciplinary literature, this perspective traces the history of the expansion of abortion access in the Americas. Mexico (2007, 2023), Uruguay (2012), Argentina (2020), and Colombia (2022) legalized abortion on demand within specific timeframes. These expansions coexist with severe restrictions on abortion in various nations including Haiti (1835), the Dominican Republic (1884, 2009), Honduras (1985, 2021), El Salvador (1997), and Nicaragua (2006), as well as some states in the United States (2022). This perspective finds that legalization occurs when feminist activists eschew U.S.-based feminist rhetoric of individual rights and choice to reframe abortion as a form of gender-based violence within a discourse of health and wellbeing as a human right. According to this perspective, restrictions on access to the procedure constitute a form of violence against women and people capable of bearing children and violate human rights.

## 1 Introduction

Latin American and the Caribbean (LAC) is a heterogeneous region comprising over 40 countries with a population of nearly 660 million people (2022) with high levels of geographic and demographic diversity. Residents speak multiple languages of European, African, and indigenous backgrounds and practice various religions (1). Since the new millennium, the region’s GDP has grown steadily, although the Great Recession and the COVID-19 pandemic caused some economic setbacks. Inequality has declined, but the region is still the second most unequal worldwide (2). Prior to the COVID-19 pandemic, the major five health indicators – life expectancy at birth and neonatal, infant (up to 12 months of age), under-5, and maternal mortality – had improved (3). Nonetheless, as Kulczycki notes (4), “significant differences in health status between and within countries” remain (p. 213) as does access to safe and legal abortion. Although abortion-related morbidity and mortality rates have decreased, they remain a public health concern, particularly in countries with restrictive bans (5).

For decades, abortion access in LAC was highly restrictive, generally correlating to a conservative Catholic stance on pregnancy interruption (6). In the United States, media representations and public opinion have contrasted this history with an allegedly more progressive national environment, where legal abortion was a constitutional right guaranteed by the Supreme Court (7, 8). But this is evidence of a U.S.-centric “coloniality of power,” in the words of decolonial scholars of Latin America (9–12). As feminist de-colonial thinkers argue, this view elevates Western modernity and rationality above formerly colonized others, who are racialized and gendered beings stuck in an imagined inferior and homogenous past (13–16).^1^ If we compare the *Dobbs v. Jackson Women’s Health Organization*’s (2022) recent overturning of *Roe v. Wade* (1973), which has allowed some states to ban the procedure, with the legalization of abortion on demand in Uruguay (2012), Argentina (2020), Mexico (2007, 2023),^2^ and Colombia (2022), and the blanket ban on abortion in more conservative countries including Haiti (1835), the Dominican Republic (1884, 2009), Honduras (1985, 2021), El Salvador (1997), and Nicaragua (2006),^3^ we see a more complicated story that challenges, as Garibotto writes (p. 686), “an ethnocentric view of the so-called South as having historically been more backward than the so-called North and an underlying assumption of Latin America as a monolithic entity” (8).

This perspective compares the simultaneous expansion and restriction of abortion access in LAC in relation to the contraction of access in large areas of the United States. It will show that in places where legalization occurred, feminist activists in LAC have reframed abortion rights within a public health framework that: (1) makes clear that the longstanding double-standard on abortion access in the region, where wealthy women access safe if clandestine procedures while poor women die from unsafe, illegal abortions, is a matter of public health and (2) that disparate access to abortion, resulting in higher rates of maternal mortality among disadvantaged women is form of violence against women and thus a violation of their human rights.

### 1.1 A brief history of abortion legislation in the Americas

The history of abortion access in the Western Hemisphere has followed a non-linear path in which criminalization and decriminalization reoccur. In the Americas, Catholic doctrine has influenced public opinion toward and legal sanctions of abortion since colonization in the early sixteenth century. In medieval and early modern Europe, most sectors of society understood abortion before quickening, or first fetal movements, as the restoration of the menses and not the intentional ending of a pregnancy. Early modern England did not criminalize the loss of a pregnancy before quickening, even if the woman^4^ or her attending midwife or physician deliberately ended the pregnancy. This understanding of when a pregnancy loss became a criminal abortion was transported to British colonies in the Americas, including what would eventually become the United States (24–26). Justice Alito’s opinion in *Dobbs* blatantly ignored this longstanding history, presenting a false past in which abortion had been criminalized since the nation’s founding (27, 28).

In medieval and early modern Iberian tradition, transferred to the Spanish and Portuguese colonies in LAC, Catholic doctrine condemned abortion as a sin, but the gravity of the act evolved significantly (29–31). Medieval Catholic theologians believed in delayed ensoulment; the fetus gained its immortal soul only after quickening. Abortion prior to quickening was a sin, but not one of murder and thus not excommunicable (32). Although increasingly questioned by theologians during the seventeenth and eighteenth centuries, this position held until the late nineteenth century, when the Church declared life as beginning at the moment of conception and all abortion, regardless the gestational age of the fetus, a sin of murder, and excommunicable (33).

Catholic doctrine coincided with Latin American independence from European colonialism in the nineteenth century, and new legislation criminalized abortion through federal penal codes (29, 34–36). In the United States, the understanding of early fetal loss as distinct from criminal abortion also shifted. In response to demographic changes, in which U.S.-born white birthrates declined as immigration and immigrant birthrates increased, nativist leaders attempted to restrict White women’s ability to regulate their fertility (37–39). Successive state-level legislation criminalized the practice in the late nineteenth century, after a professionalizing American Medical Association began a nationwide campaign to crack down on pregnancy termination at all gestational stages (37, 39).

Despite this trend toward criminalization, Brazil and Argentina were some of the first countries in the world to legalize therapeutic abortions in the early twentieth century (34, 35, 40), although they lacked adequate protocols to ensure access to legal procedures. In Brazil – which legalized therapeutic abortions in the late nineteenth century if the mother’s life was in danger, and in 1940 expanded this legislation to include cases of rape – medical regulations related to health provisions only appeared in the 1920s (35). The government finally issued regulations regarding rape in 1999 (updated in 2012) and for all reasons in 2005 (41, 42). Argentina criminalized abortion in 1921 except in cases of the risk to the health or life of the mother or in cases of rape (43). As late as 2019, only 10 out of 24 jurisdictions in Argentina had up-to-date medical protocols (44).

By the early twentieth century, abortion was illegal in most of the United States and Latin America and the Caribbean. This hardened approach toward the voluntary ending of a pregnancy remained stable, if not enforceable, until the second half of the twentieth century. For on-demand abortions, the U.S. broke this trend with the passage of *Roe* in 1973. In LAC, Cuba became the first country to legalize on-demand abortion, providing all women free access to the procedure in 1979 (45, 46). In 1988, Canada legalized on-demand abortion and further stipulated the government provide services free of charge under the Canada Health Act (47).

As some nations began decriminalizing abortion, the topic entered the public sphere in LAC, with the better recording and publication of health complications and deaths related to illegal procedures. In Brazil, studies in the 1980s found that poor women, often women of color, disproportionately experienced higher rates of maternal mortality and morbidity related to unwanted pregnancy (48). Although data are incomplete, experts have hypothesized that abortion played an important role in Latin America’s overall fertility decline beginning in the 1960s and continuing through the 1980s, until more widespread use of biomedical contraceptives became prevalent (4, 49).

As maternal deaths from abortion became visible, second-wave feminists began organizing around abortion rights (50–52). Many of these feminists lived in countries under authoritarian dictatorships that violently suppressed political dissent. Questions of bodily autonomy thus had broader meanings during the Cold War in Latin America: what did abortion access imply if a military government could “disappear” (kidnap, torture, and murder) one’s family members without consequence? Latin American feminists’ demands to legalize abortion thus began as part of a human rights narrative in which bodily autonomy for all citizens was a fundamental aspect of redemocratization (53, 54).

Due to this historical context, feminist organizing in Latin America for reproductive health equity had always employed a reproductive justice framework, linking abortion rights to economic and social justice (43, 44, 55, 56). Reproductive justice, according to the Black U.S. feminists who coined the term in the mid-1990s, is a human-rights based theory and praxis of reproductive autonomy in which all people have the “right to maintain personal bodily autonomy, have children, not have children, and parent the children we have in safe and sustainable communities” (57). Yet only the most marginalized in the U.S. – Black, indigenous, and other women of color – have harnessed international human rights laws and discourses framework to fight against a racist and patriarchal state that disregards human life within their communities (28, 58–62). Mainly White feminist organizations focused on choice and the negative right of privacy at the expense of a broader understanding of how abortion access fits into human rights, including the positive right of access to healthcare (38, 63, 64).

### 1.2 Ready for change

Since the new millennium, feminists fighting for reproductive justice including abortion rights and an end to obstetric violence in LAC have reframed any restriction on abortion as an act of gender-based violence and thus as a human rights violation (53, 54, 65–68). In the 1990s and 2000s, legislative successes to decriminalize or legalize abortion in LAC were few and existed alongside newly enacted blanket bans. El Salvador criminalized abortion under all circumstances in 1998, which has had severe consequences not only for women’s lives, as poor women face life-and-death situations when accessing abortion care, but also for gender equality (69). At the same time, the continued activism of feminists and other civil society actors allowed for modest reforms in Mexico, Brazil, and Colombia, which set the stage for more recent changes (4).

In the past 15 years, this human rights foundation has proven crucial for pushing legislative successes forward. Feminists organizing to legalize abortion in Argentina have eschewed discussions of when life begins or privacy rights, instead emphasizing social equality and economic justice (56). Arguing that Argentine women from all classes have abortions, but only poor women die from them, feminists moved the conversation away from the morality of abortion – women need the procedure regardless of its legality – to the inequity of differential health outcomes based on social class (70). They also incorporated demands for safe and legal abortion within larger social justice campaigns, thus expanding their base of supporters (43, 44). Feminists tied longstanding public health arguments in favor of decriminalizing abortion to calls against gender-based violence, likening maternal deaths from abortion to femicides. Following a reproductive justice framing, the bill that legalized abortion in Argentina requires that public hospitals provide the procedure free of charge (71). The final bill also includes gender-inclusive language by allowing access to abortion on demand up to 14 weeks for “women and all gestating persons” (43).

An economic justice argument was also crucial in the legalization of abortion in Colombia in 2022 (53). Strategic litigation activities in the first two decades of the new millennium culminated in the Constitutional Court’s passage of one of the world’s broadest on-demand abortion laws (72). In Brazil, feminists are pushing the Supreme Court to decide whether the criminalization of abortion violates the human rights of “women, adolescents or girls” (73). As of September 2023, the Brazilian Supreme Court is currently debating the decriminalization of abortion up to 12 weeks LMP on these grounds (74). In September 2023, the Mexican Supreme Court declared all criminal penalties for abortion unconstitutional, stating that they “violate the human rights of women and people with the ability to gestate” (75). A second decision issued only days later made it unconstitutional to define legal personhood as “from conception.” It also later invalidated conscientious objection by physicians and other medical practitioners. This decision built upon one two years prior that recognized the constitutional right to free abortion services up to 12 weeks LMP and on specific grounds after that time frame in the Mexican state of Coahuila (76).

Shifting religious patterns also has affected public opinion; Catholic religiosity has declined across the region, and despite a rising percentage of Latin Americans who identify as evangelicals, overall church attendance is down (44, 53). Nonetheless, the Catholic church has proven a stalwart against abortion rights, and since the 1990s, it also has employed the language of human rights in its pro-life efforts (7). A growing, vocally anti-abortion evangelical movement has joined these endeavors (4, 77). In the U.S., pro-life evangelicals successful restricted abortion access in the decades leading up to the overturning of *Roe* (78, 79). This tactic must be seen as a threat to the viability of any on-demand abortion policy. In the U.S., increasing restrictions at the state level after *Planned Parenthood v. Casey* (1992), severely constrained abortion access for many pregnant people, particularly the most marginalized, by shifting the legal context to an “undue burden” standard (80). As abortion providers and pregnant people know best, legal abortion does not equal accessible abortion services (71, 80–82). For example, the September 2023 Mexican Supreme Court decriminalization of abortion does not automatically equate to full access. As Valero writes, “For decriminalization to really translate into a future with greater access to reproductive health care, abortion-seekers need access to psychological and social support, as well as clinics and hospitals stocked with essential drugs, supplies, equipment, and trained specialists” (83).

## 2 Discussion

Social science scholars have urged U.S. feminists to reframe their fight for abortion rights by drawing on Latin American successes. Fixmer-Oraiz and Murillo argue that U.S. feminists must name “abortion care denial as *violence*” (54). In the U.S., political culture is strongly focused on individual rights and decision making. This includes a Malthusian approach toward population politics, in which the poor are blamed for allegedly irrational choices, including to get an unsafe abortion. Such exceptionalist discourses exist on both the left and the right, underscoring the rejection of international frameworks including those supporting human and women’s rights. Given this, and in the face of extreme efforts to criminalize reproductive choices in the U.S., how can we implement this necessary shift?

Pregnant people will always need access to abortion services regardless the legal restrictions or permissions. To reframe abortion as a public health and thus human rights issue, we must move away from all discussions tied to fetal personhood, which the pro-life movement has successfully operationalized to criminalize abortion. The time parameters for on-demand abortion do not necessarily need to extend until fetal viability. *Roe* marked viability as the end of on-demand abortion. Most Latin American nations that recently legalized abortion on demand have implemented shorter gestational limits. To delink fetal viability and on-demand abortion, two conditions must be met: (1) pregnant people must remain able to terminate pregnancies for expansive health-related issues after on-demand gestational limits end and (2) on-demand abortion access must be available to everyone, not just to those who can afford it, so that people who need an abortion can get it early in the pregnancy. On-demand abortion must be tied to public health calls for broader universal health services and the even distribution of quality reproductive healthcare across the nation. The relevant personhood is that of the pregnant person, not the fetus. So, in this sense, taking one part of LAC platform – that inequity in healthcare is gender-based violence – we can push forward to expand universal healthcare and abortion access.

This reframing must also exist alongside another successful trend coming from LAC: grassroots “accompanist networks” that have created extensive cross-regional networks to provide mainly medication abortion to women who live in areas with restrictive laws (84–86). Pregnant people have long travelled to access abortion, but this travel reinforces economic barriers (87, 88). In the U.S., abortion funds have stepped into this role, providing funding for travel and abortion care in highly restricted contexts. Often deeply embedded within local activist networks, these groups are the collective grassroots organizing that must thrive while policymakers and public health practitioners advance abortion access on the population level. But an initial outpouring of donations in the year following Dobbs has begun to dry up (89), and experts have urged donors to “take the long view” (90).

In the U.S., the rise of self-managed abortions has allowed pregnant people to obtain prescriptions for the drug combination of mifepristone and misoprostol for medication abortions at home (91, 92). Pharmacists in Brazil began providing misoprostol (an ulcer drug) off label to women who wanted to terminate their pregnancies in the 1990s (93–95). Today, accompanist networks in many parts of Latin America have expanded access to medication abortions by providing pregnant people living in restrictive legal contexts misoprostol for self-managed abortions with safety guidelines and support (84, 96–100). Feminist networks are already doing the same in the United States (92), although not without legal consequences (101). As we engage in the crucial work of re-expanding abortion access, we cannot forget the pregnant people who need an abortion right now.

## Data availability statement

The original contributions presented in the study are included in the article/Supplementary Material, further inquiries can be directed to the corresponding author.

## Author contributions

CR: Conceptualization, Investigation, Methodology, Project administration, Writing – original draft, Writing – review & editing.

## Footnotes

^1^Here, I focus on critical decolonial theory coming from Latin America, but other relevant theoretical models include Stuart Hall’s discussion of representation (17) and postcolonial theories of Otherness (18, 19), among others. Bhambra (20) has an excellent synthesis of the two traditions.

^2^Mexico City decriminalized abortion up to 12 weeks LMP in 2007. In 2021, the country’s Supreme Court declared the criminalization of abortion up to 12 weeks LMP in the state of Coahuila unconstitutional. In 2023, it expanded that decision to all Mexican states.

^3^Haiti, the Dominican Republic, and Honduras banned abortion in all circumstances in 1835, 1884, and 1985, respectively. The Dominican Republic and Honduras, in 2009 and 2021 respectively, wrote these bans into their constitutions. Abortion also is completely banned in Jamaica, Suriname, Curaçao, and Aruba (21). In 2020, Haiti passed a new Penal Code that would have legalized abortion on demand up to 12 weeks LMP, but the president was assassinated, and the Code has not been implemented (22).

^4^Until the late twentieth century, legal and medical sources, as well as people, used the term “women” or “mother” in relation to abortion care. This article adheres to this terminology for historical accuracy. However, its discussion of contemporary activism and policies employs the more gender-inclusive language of pregnant people (23).
